# White Matter Tracts in Patients with Temporal Lobe Epilepsy: Pre- and Postoperative Assessment

**DOI:** 10.7759/cureus.1735

**Published:** 2017-10-01

**Authors:** Fateme Salehi, Manas Sharma, Terry M Peters, Ali R Khan

**Affiliations:** 1 Department of Radiology, Western University; 2 Department of Radiology, Western University; 3 Medical Biophysics, Western University; 4 Medical Biophysics, Western University

**Keywords:** temporal lobe epilepsy, mesial temporal sclerosis, tractography, temporal lobectomy

## Abstract

Patients with intractable temporal lobe epilepsy (TLE) undergo surgical resection of the anterior temporal lobe. Preoperative assessment of TLE patients involves a multidisciplinary assessment and may involve the use of invasive electroencephalogram (EEG) recording for lateralization of seizure focus in ambiguous cases. Understanding the white matter fibre tracts affected in TLE may assist in preoperative lateralization and planning. We studied pre- and postoperative white matter fibre tract changes in six patients with TLE who underwent surgical resection. Our results indicate that changes in the corpus callosum are highly specific, with the ability to lateralize the epileptogenic side in 100% of our patients (six of six). Contralateral changes were found in all patients with variable involvement of white matter tracts. Postoperatively, most patients (five of six) exhibited further changes to the tracts on the ipsilateral side, with three patients showing contralateral abnormalities. We provide a detailed assessment of pre- and postoperative white matter fibre tracts in patients with TLE and confirm that abnormalities in the ipsilateral corpus callosum may aid in preoperative lateralization and obviate the need for invasive EEG monitoring.

## Introduction

Approximately 20% of patients with temporal lobe epilepsy (TLE) are refractory, exhibiting no response to anti-epileptic drugs [1]. A subset of these patients with refractory epilepsy are potential candidates for surgical resection, mostly an anterior temporal lobectomy with amygdalohippocampectomy. Resection of the anterior temporal lobe is an effective and increasingly used treatment for refractory medial temporal lobe epilepsy [2-3]. Preoperative assessment of patients with TLE requires extensive evaluation by a team of neurologists, neurosurgeons, neuroradiologists, and neuropsychologists. Even after extensive imaging and clinical and scalp electroencephalogram (EEG) assessment, the epileptogenic focus remains elusive in some patients who then undergo invasive EEG monitoring, mostly with the placement of subdural electrodes. Invasive monitoring can involve surgical complications and postoperative deficits, in addition to the further delay of surgical resection [4-5]. Facilitating a non-invasive preoperative diagnosis is essential. Diffusion tensor imaging (DTI) and tractography allow for evaluation of white matter fibre tract integrity and composition that is not fully evaluated by structural magnetic resonance imaging (MRI). Studies have demonstrated ipsilateral white matter changes in patients with TLE, which may facilitate lateralization [6]. Therefore, we investigated a large number of tracts in patients pre- and postoperatively to improve the current understanding of white matter tract abnormalities and the ability to provide additional lateralizing information [6].

## Case presentation

This study was approved by our Human Resources Ethics Committee via Institutional Review Board. The study included six patients with a history of epilepsy resistant to anti-seizure medication (average age: 40.3, range: 23 - 58). Clinicopathological data are summarized in Table 1. There were four female and two male subjects. The patients underwent 1.5 Tesla (1.5T) and 3 Tesla (3T) magnetic resonance imaging (MRI) for evaluation of epilepsy as a part of our standardized comprehensive epilepsy evaluation. Video- electroencephalogram (EEG) and multidisciplinary assessment had confirmed seizure onset in the mesial temporal lobe (MTL) ipsilateral to the clinically defined seizure site. In four of the six patients, structural MRI scans demonstrated mesial temporal sclerosis. All patients had a normal contralateral hippocampus based on the preoperative MRI. All patients were taking anti-epileptic medications. Patient handedness and language dominance were determined by neuropsychological testing. Patient demographics and clinical information are summarized in Table 1.

**Table 1:** Patient Clinicopathological Information *Generalized supratentorial atrophy; EEG: electroencephalography; MRI: magnetic resonance imaging; R: right; L: left; RATL: right anterior temporal lobectomy; LATL: left anterior temporal lobectomy; MTS: mesial temporal sclerosis

Patient number	Age	Gender	Handedness/ Language Dominance	Age of Onset	Clinical and EEG Diagnosis	Preop MRI summary (1.5T)	Type of Surgery	Hippocampus/Amygdala Pathology
1	56	F	R	15	R	Atrophy*	RATL	Gliosis
2	43	F	R	3	R	R MTS	RATL	MTS
3	23	M	R	18	L	Normal	LATL	Gliosis
4	34	M	L	15	L	L MTS	LATL	MTS
5	58	F	R	2	R	R MTS	RATL	MTS
6	28	F	R	23	L	L MTS	LATL	MTS

We performed whole-brain tractography using the BrightMatter™ System (Synaptive Medical, Toronto, Canada) neurosurgical planning system, which performs automated processing of T1-weighted anatomical images and diffusion-weighted images to perform skull-stripping, co-registration, diffusion tensor fitting, and whole brain tractography. Specific bundles were assessed through 1) modulating the tract filtering slide bars, 2) displaying tracts in specific orthogonal planes, and 3) filtering tracts according to the intersection with a surgical trajectory.

Qualitative assessment was performed by the study author (FS) assessing reconstructed tracts on superimposed three-dimensional (3D) T1-weighted images and the methodology was verified by a neuroradiologist (MS). The anatomic course of each fibre was verified in axial, coronal, and sagittal planes. The healthy side was compared to the contralateral epileptogenic side. The preoperative and postoperative images and tracts were also evaluated for differences. In the postoperative patients, thinning was documented if there was additional thinning compared to the preoperative images only. Tract thinning between hemispheres was assessed by first isolating the tract using appropriate coronal or axial slices, then sweeping the tract reduction (thresholding) sliders across their extents, while assessing whether any asymmetries exist. 

Assessment parameters included macroscopic white matter tract dislocation or thinning. The abnormalities were documented in the ipsilateral and contralateral sides in the preoperative and postoperative images and compared.

All patients underwent surgical resection following planning by a multidisciplinary team of epilepsy neurologists and neurosurgeons. Mesial temporal resection included the amygdala and anterior part of the hippocampus. Postoperative pathology was concordant with preoperative MRI findings in four patients with mesial temporal sclerosis (MTS). Two patients demonstrated non-specific gliosis.

The white matter tracts evaluated for this study are the corpus callosum (CC), fornix (F), cingulum (C), superior longitudinal fasciculus (SLF), inferior longitudinal fasciculus (ILF), arcuate fasciculus (AF), uncinate fasciculus (UF), inferior fronto-orbital fasciculus (IFOF), optic radiations (OR), corona radiata (CR), and corticospinal tracts (CST). Abnormalities in preoperative studies included thinning of white matter tracts on the side ipsilateral to the epileptogenic side (six patients), the contralateral side (six patients), or bilateral abnormalities (six patients) were appreciated in some tracts as specified below. The details are recorded in Table 2. Postoperative abnormalities included further thinning and disruption of the white matter tracts in the ipsilateral side (five patients), the contralateral side (three patients), or bilateral abnormalities (three patients). The results are summarized in Table 2, reflecting the total number of patients with tract thinning.

**Table 2:** Pre- and Postoperative Findings Showing the Number of Tracts with Thinning In postoperative patients, thinning was documented if there was additional thinning compared to the preoperative images only. CC: corpus callosum; F: fornix; C: cingulum; SLF: superior longitudinal fasciculus, ILF: inferior longitudinal fasciculus; AF: arcuate fasciculus; UC: uncinate fasciculus; CST: corticospinal tracts; IFOF: inferior fronto-orbital fasciculus; OR: optic radiations; CR: corona radiata

	Preoperative Thinning			Additional Postoperative Thinning		
Tracts	Ipsilateral	Contralateral	Bilateral	Ipsilateral	Contralateral	Bilateral
CC	6	0	0	2	1	1
F	5	0	0	2	0	0
C	5	3	2	2	0	0
SLF	4	2	0	3	1	1
ILF	4	2	0	1	0	0
AF	3	1	0	2	0	0
UF	5	1	0	2	1	1
CST	5	0	0	1	0	0
IFOF	1	3	0	0	0	0
OR	3	1	0	4	0	0
CR	3	1	0	4	1	1

**Figure 1:**
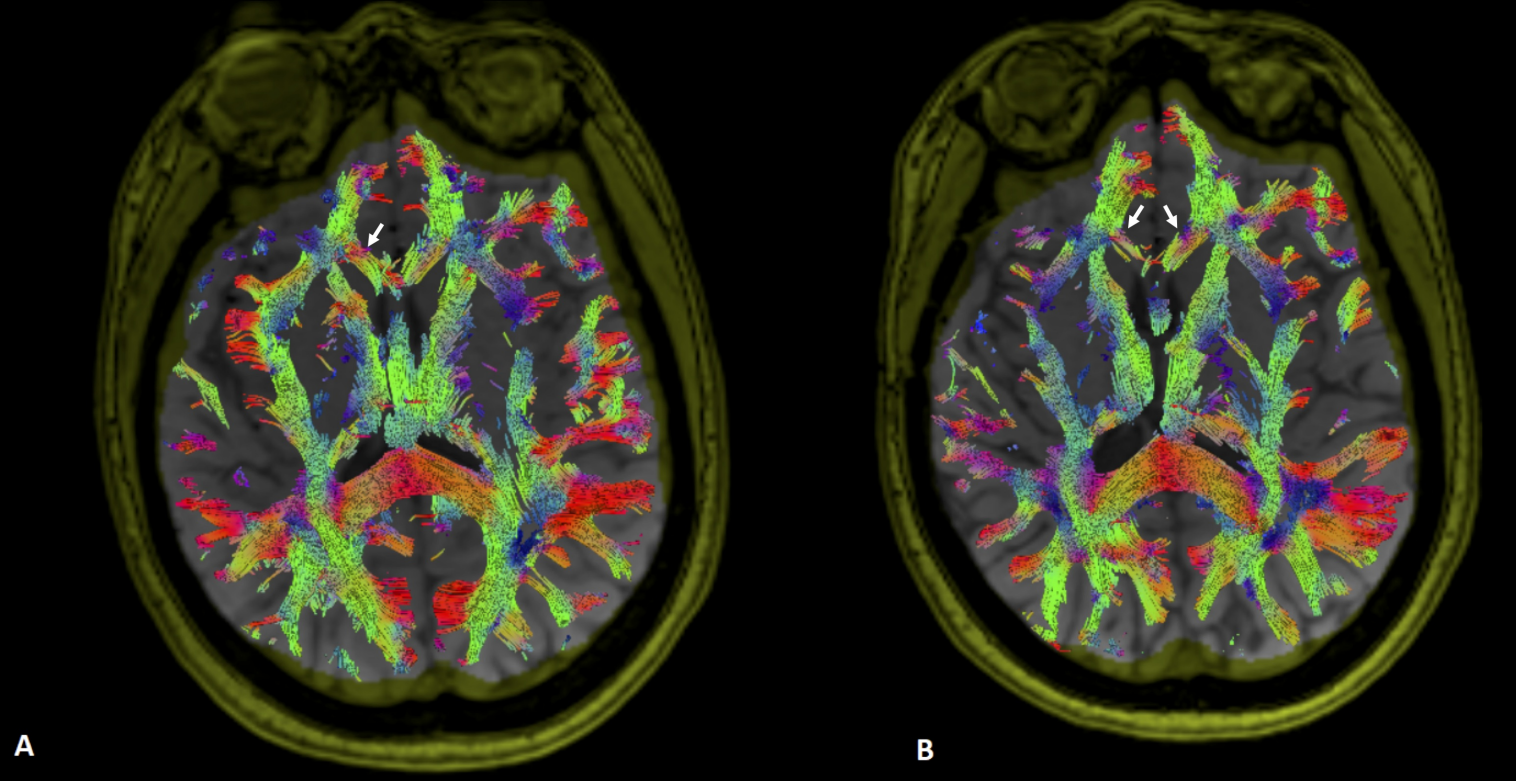
Comparison of pre- and postoperative changes in deep white matter tracts in a patient with right temporal lobe epilepsy. A) Preoperative diffusion tensor fibre tracking of the corpus callosum in a patient with right mesial temporal sclerosis and epilepsy is shown overlaid on T1-weighted axial magnetic resonance imaging (MRI). Preoperatively, the corpus callosum fibres within right minor forceps demonstrate thinning compared to the left (arrow); B) Tracts were analyzed in the same patient after anterior temporal lobe resection. Diffusion tensor fibre tracking exhibits further thinning of the tracts within the minor forceps of the corpus callosum ipsilateral to the resection side (right arrow). Additionally, the left forceps minor demonstrates increased thinning of tracts not seen preoperatively.

All six patients exhibited thinning and disruption of the corpus callosum in the ipsilateral side with no documented abnormalities in the contralateral side (Figure 1). Additionally, five of the six patients demonstrated abnormalities in the ipsilateral fornix without any contralateral abnormalities. The only tract with bilateral preoperative abnormalities was the cingulum (two patients). In the case of cingulum, thinning of fibers at the anterior inferior cingulum was confined to the ipsilateral side, although the contralateral side demonstrated thinning of the posterior fibers in two patients. The remainder of the tracts demonstrated either ipsilateral or contralateral thinning and disruption. The ipsilateral UF and CSTs were abnormal in five patients. Four patients had ipsilateral superior and inferior longitudinal fasciculus abnormalities. Three patients demonstrated abnormalities in AF, OR, and CR. One patient had ipsilateral IFOF changes. Contralateral abnormalities were more numerous in IFOF than other tracts (three patients).

**Figure 2:**
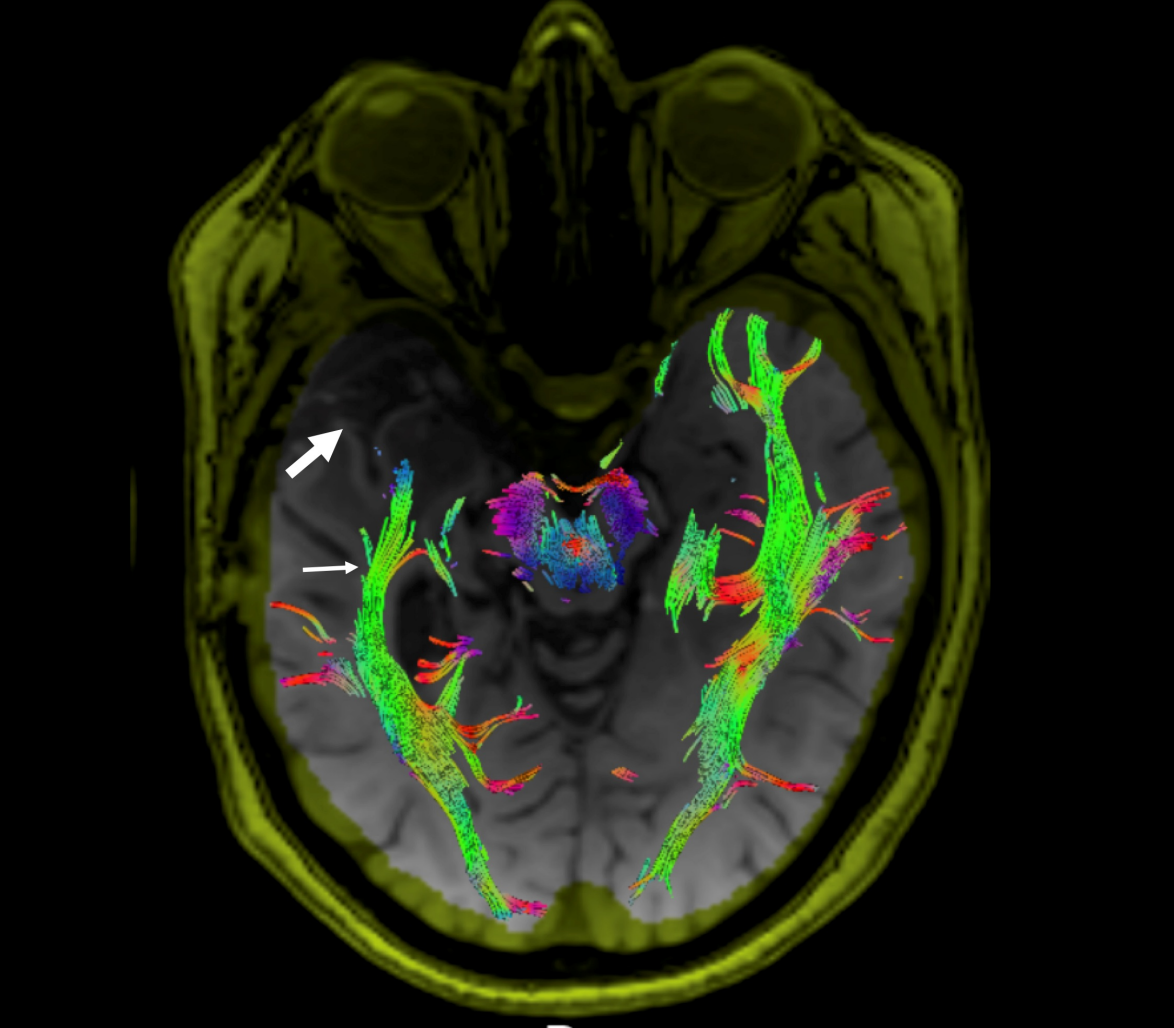
Diffuser tensor imaging and tractography of the optic radiations in a case of unilateral mesial temporal sclerosis (MTS) following resection of the right anterior temporal lobe. The optic radiations are depicted by using tractography. The tracts are overlaid on T1-weighted images. Postoperative changes, including encephalomalacia, are demonstrated within the right middle cranial fossa and the surgical site is denoted by the large arrow. The right optic radiations are thinned and disrupted on the ipsilateral side (small arrow).

New or further thinning and disruption of ipsilateral white matter tracts were present in four patients. Table 2 documents only new findings in the postoperative group, with baseline preoperative thinning not included in the postoperative group numbers. The most affected tracts were OR and CR (four patients), followed by SLF (three patients) (Figure 2). Contralateral abnormalities were noted in the CC (one patient), SLF (one patient), UF (one patient), and CR (one patient). Bilateral abnormalities were present in CC (one patient) and CR (one patient).

## Discussion

Recent DTI studies have demonstrated abnormalities within several white matter tracts in patients with TLE [6]. The current study extends the literature by demonstrating the widespread changes in the white matter tracts preoperatively. We also examined white matter fiber changes in patients after surgical resection and evaluated changes occurring postoperatively.

We evaluated six patients with TLE who underwent resection of the mesial temporal lobe for drug-resistant epilepsy. Fiber tract thinning and asymmetry was found in both the ipsilateral and contralateral sides to the seizure focus. Assessment of pre- and post-surgical white matter fiber bundles by MR imaging tractography revealed abnormalities in all 11 tracts studied. Pre-surgically, all patients demonstrated asymmetry in the white matter tracts within their ipsilateral corpus callosum, with most patients (five of six) exhibiting ipsilateral changes in the fornix, cingulum, uncinate process, and the CSTs. The strong association of ipsilateral thinning of the corpus callosum fibers, mainly along with the described abnormalities, may provide further lateralizing information and aid in the determination of the epileptogenic side. Tractography may serve as an additional tool in the preoperative assessment of patients in whom the epileptogenic focus is indeterminate and may obviate the need for invasive EEG recording, including subdural and depth electrodes.

Our findings are in keeping with previous studies that showed abnormalities in the cingulum, fornix, and corpus callosum to provide distinctive diffusion indices in patients with TLE with the ability to distinguish the pathological side [7]. Nazem-Zadeh, et al, using DTI characteristics of various brain regions to differentiate between left, right, and bilateral TLE patients, found that differences in the corpus callosum, fornix, and cingulum were the most specific abnormalities for lateralization. In our series, all patients demonstrated ipsilateral abnormalities in the corpus callosum without contralateral changes, confirming the specificity of corpus callosum abnormalities for lateralization of patients with epilepsy. Similarly, five patients who demonstrated ipsilateral thinning in the fornix had no contralateral changes, making the use of fornix tract abnormalities highly specific for detection of lateralization. In the case of the cingulum, thinning of fibers at the anterior inferior cingulum was confined to the ipsilateral side, although the contralateral side demonstrated thinning of the posterior fibers in two patients. Our results are concordant with a previous study by Nazem-Zadeh, et al. [7]. These findings support the use of tractography in patients without identifiable hippocampal abnormality and in whom the seizure focus is ambiguous. As an additional tool in the preoperative assessment of patients with epilepsy, tractography can delineate anatomical asymmetries that may contribute to functional reorganization noted in patients with TLE, facilitate lateralization of the epileptogenic side, and potentially obviate the need for invasive EEG recording.

Postoperative assessment of the white matter fiber tract showed the progression of preoperative abnormalities or new fiber tract abnormalities, most prominently in the OR and CR, as expected. These fibers course through the temporal lobe and surgical resection impacts the anatomy and function. Ipsilateral abnormalities in postoperative patients can be directly attributed to resection of the surgical lesion. The abnormalities were not, however, limited to the ipsilateral temporal lobe, concordant with previous findings of widespread changes in TLE patients who undergo resection [8]. Degradation of fibers was more frequent in the ipsilateral hemisphere; however, three patients demonstrated new asymmetry and fiber degradation on the contralateral side involving the CC, UF, and CR. As expected, fiber tracts connected to the resected hippocampus undergo Wallerian degeneration and postoperative thinning and disruption. On the contralateral side to seizure focus and resection, changes were present in the UF, which connects the anterior temporal lobe with medial and orbital prefrontal cortex in a bidirectional manner [9]. The UF plays an important role in episodic memory formation and retrieval and changes may result in impaired memory function postoperatively.

Investigators have found more widespread contralateral and bilateral abnormalities in patients with left TLE, with more ipsilateral abnormalities in patients with right TLE [6]. We found that in the three patients with left TLE, bilateral abnormalities were observed in seven of the 11 tract studies. Similarly, in the three patients with right TLE, seven tracts exhibited bilateral abnormalities. The lack of difference in the two subgroups may be due to the small number of patients included in this study.

The corpus callosum is a major interhemispheric tract that is essential in cognitive functions, and previous DTI studies have reported a high specificity for asymmetrical interruption of ipsilateral corpus callosum for lateralization of epilepsy. In our series, only one patient demonstrated changes to the contralateral corpus callosum postoperatively. The connections that exist between the corpus callosum and the temporal lobe may contribute to these postoperative abnormalities. Fiber tracts that originate from hippocampal formation and amygdala, which are involved in epileptogenesis and connections to other ipsilateral and contralateral anatomical structures, play an essential role in spreading neuronal activity during seizures and are altered in patients with TLE. Recently, reorganization of language tracts in the contralateral non-dominant hemisphere following resection of the anterior temporal lobe has been demonstrated [10], in keeping with our findings of contralateral changes in white matter tracts.

## Conclusions

This small case series suggests the potential for the specificity of corpus callosum thinning in the ipsilateral side to the epileptogenic focus in patients with TLE for lateralization. All patients exhibited ipsilateral thinning and disruption of the corpus callosum preoperatively compared to the contralateral side. Therefore, preoperative whole brain tractography may be considered as an additional tool to complement EEG and clinical assessment in cases with an ambiguous seizure side, potentially obviating the need for invasive EEG recordings. We also demonstrated bilateral changes in pre- and postoperative patients, in keeping with the diffuse connectivity network involved in temporal lobe epileptogenesis and propagation. Further studies are underway to include inter-rater reliability assessment and correlation with quantitative evaluation.
